# Evaluating in vivo efficacy – toxicity profile of TEG001 in humanized mice xenografts against primary human AML disease and healthy hematopoietic cells

**DOI:** 10.1186/s40425-019-0558-4

**Published:** 2019-03-12

**Authors:** Inez Johanna, Trudy Straetemans, Sabine Heijhuurs, Tineke Aarts-Riemens, Håkan Norell, Laura Bongiovanni, Alain de Bruin, Zsolt Sebestyen, Jürgen Kuball

**Affiliations:** 1 Department of Hematology and Laboratory of Translational Immunology, University Medical Center Utrecht, Utrecht, The Netherlands; 2 Faculdade de Medicina, Instituto de Medicina Molecular, Universidade de Lisboa, Lisbon, Portugal; 3 Department of Pathobiology, Faculty of Veterinary Medicine, Dutch Molecular Pathology Center, Utrecht University, Utrecht, The Netherlands

**Keywords:** AML, Immunotherapy, TEGs, Mice model, Preclinical, Toxicity, TCR engineering

## Abstract

**Background:**

γ9δ2T cells, which express Vγ9 and Vδ2 chains of the T cell receptor (TCR), mediate cancer immune surveillance by sensing early metabolic changes in malignant leukemic blast and not their healthy hematopoietic stem counterparts via the γ9δ2TCR targeting joined conformational and spatial changes of CD277 at the cell membrane (CD277J). This concept led to the development of next generation CAR-T cells, so-called TEGs: αβT cells Engineered to express a defined γδTCR. The high affinity γ9δ2TCR clone 5 has recently been selected within the TEG format as a clinical candidate (TEG001). However, exploring safety and efficacy against a target, which reflects an early metabolic change in tumor cells, remains challenging given the lack of appropriate tools. Therefore, we tested whether TEG001 is able to eliminate established leukemia in a primary disease model, without harming other parts of the healthy hematopoiesis in vivo.

**Methods:**

Separate sets of NSG mice were respectively injected with primary human acute myeloid leukemia (AML) blasts and cord blood-derived human progenitor cells from healthy donors. These mice were then treated with TEG001 and mock cells. Tumor burden and human cells engraftment were measured in peripheral blood and followed up over time by quantifying for absolute cell number by flow cytometry. Statistical analysis was performed using non-parametric 2-tailed Mann-Whitney t-test.

**Results:**

We successfully engrafted primary AML blasts and healthy hematopoietic cells after 6–8 weeks. Here we report that metabolic cancer targeting through TEG001 eradicated established primary leukemic blasts in vivo, while healthy hematopoietic compartments derived from human cord-blood remained unharmed in spite of TEGs persistence up to 50 days after infusion. No additional signs of off-target toxicity were observed in any other tissues.

**Conclusion:**

Within the limitations of humanized PD-X models, targeting CD277J by TEG001 is safe and efficient. Therefore, we have initiated clinical testing of TEG001 in a phase I first-in-human clinical trial (NTR6541; date of registration 25 July 2017).

**Electronic supplementary material:**

The online version of this article (10.1186/s40425-019-0558-4) contains supplementary material, which is available to authorized users.

## Background

Adoptive cell therapy with engineered immune cells targeting hematological malignancies entered clinical practice [1]. Reprogramming immune cells has been achieved so far with chimeric antigen-reactive receptors [2] and tumor-specific αβT cell receptors (TCRs) [3, 4]. However, the CAR-T concept frequently targets ubiquitously expressed antigens like CD19 for B cell malignancies [5], or FLT-3 [6] for acute myeloid leukemia (AML), as well as stress antigens like NKG2D (natural-killer group 2, member D) for a broader range of cancers [7], raising the question of whether such strategies result as collateral damage in either the long-term deletion of essential hematopoietic subsets or within the context of physiological or therapeutic stress like irradiation to self-reactivity. Given the low mutational load of AML [8], the targeting of neo-antigens has not been successful, and targeting of minor antigens like HA-1 allows only the inclusion of a minority of patients [9]. Thus, novel strategies are needed to attack myeloid malignancies within the context of engineered immune cells.

One very attractive and so far, not well-explored alternative to mediate tumor-specific TCR derives from unconventional γ9δ2T cells subsets [10]. γ9δ2T cells sense molecular stress signatures via the accumulation of intracellular phosphoantigens level on infected and malignant cells [11]. This cell subset has the ability to kill tumor cells originating from hematological and solid malignancies in vitro, making it a promising immunotherapeutic option [10, 12]. While several clinical trials have been conducted using ex vivo expanded and adoptively transferred autologous γ9δ2T cells in patients with advanced malignancies including AML, the results showed scarce activity [13]. One major obstacle has been the limited proliferative capacity of γ9δ2T cells in advanced cancer patients [14], as well as the underestimation of the substantial molecular and functional diversity within this subset [15, 16]. Therefore, alternative strategies are needed for the clinical translation of the strong antitumor reactivity of receptors expressed on γ9δ2T cells [15].

To override the major weakness of γ9δ2T cells for its defective proliferative capacity and underestimated diversity, our group demonstrated that αβT cells engineered to express a defined γδTCR, so-called TEGs, solves the proliferation deficiency and diversity of γ9δ2T cells by utilizing one defined γ9δ2TCR with strong antitumor reactivity and the strong proliferative capacity of αβT cells. Furthermore, by utilizing αβT properties, we retain both CD4^+^ and CD8^+^ effector cell functions in our TEGs. The first clinical candidate of TEGs derived from clone 5 (TEG001) has been shown to mediate the highest antitumor reactivity against a broader panel of tumor cells in vitro and in cell line-derived xenograft mouse models and to outperform natural γ9δ2T cells [10, 12, 17, 18]. However, the assessment of the true activity of TEG001 against primary leukemia as well as potential toxicity in physiologically more relevant models has not been assessed so far, but is essential prior to entering a first-in-human clinical trial. Low toxicity of natural γ9δ2T cells in many clinical trials [13] cannot be used as an argument for safety, given also their lack of activity in men, mainly orchestrated through many NK-like immune inhibitory receptors expressed at the cell surface of natural γ9δ2T cells [16]. The major driver of the activity of TEG001, but also its potential risk of toxicity, is derived from the concept of utilizing a highly active γ9δ2TCR out of the context of the natural brakes of a γ9δ2T cells, which have been also the pitfalls for their successful clinical translation to date. Thus, the key obstacle of clinical translation of TEG001 remains the assessment of its bare activity against primary leukemia as well as potential side effects against e.g. healthy hematopoietic compartments. Classical concept of efficacy and safety testing fail for this novel type of tumor-specific antigen, given that a joint conformational and spatial change of CD277 (later referred to as CD277J) mediated through early metabolic changes in cancer cells is recognized by the utilized γ9δ2TCR [12, 19, 20] and no tools are available to directly assess CD277J. To date, only cellular re-localization of RhoB can serve as a surrogate marker of CD277J [12]. Antibodies used for detecting CD277 rather induce or inhibit the conformational and spatial changes of CD277J [21, 22], thus they do not have the intrinsic ability to sense these alterations. Soluble γδTCR have been suggested to sense CD277J [23], however a more comprehensive analysis of such tools could not confirm their suitability, most likely due to the low affinity of the γ9δ2TCR (J Kuball unpublished observation). To remove these obstacles before clinical testing, we developed models which allow us to assess efficacy and toxicity of TEG001 in more physiological relevant environments, with primary tumor tissues as well as primary healthy cells. One example is the recently established 3D bone marrow model which enabled us to determine the efficacy of TEG001 against primary multiple myeloma cells, and to simultaneously exclude toxicity against stroma and endothelial cells in the bone marrow niche [24]. However, limited information is available when assessing activity of TEG001 against established leukemia, and toxicity against the complete hematopoietic compartment. Therefore, we utilized in this report an in vivo patient-derived xenograft (PD-X), and a healthy donor-derived xenograft (HD-X) model for assessing the efficacy of TEG001 against primary leukemic blasts and toxicity against the complete hematopoietic compartment, to provide a rationale for first-in-human testing of TEG001.

## Materials & methods

### Functional T cell assay

IFNγ ELISPOT was performed using anti-human IFNγ mAb1-D1K(I) and mAb7-B6–1 (II) (Mabtech) in accordance with the manufacturer’s protocol. Effector and target cells (E:T 1:3) were incubated for 24 h with or without pamidronate (10 or 100 μM, Calbiochem) as indicated. Pamidronate was added in all our in vitro experiment in order to enhance TEGs activation as previously reported [10].

### RhoB distribution analysis using confocal microscopy

Human CD34^+^ progenitor cells from a healthy donor were subjected to different conditions as follows: 1) untreated; 2) overnight stimulation with 50 IU/ml IL-2 or 3) 1000 IU/ml IFNγ; 4) overnight incubation in the presence of 5 mM Cyclophosphamide (Cy, Sigma-Aldrich Chemie NV, South Holland), or 5) 20 μM Fludarabine-phosphate (Flu, Sigma-Aldrich Chemie NV, South Holland), or 6) Cy/Flu combination. Primary AML, B cells, T cells, and monocytes were exposed to 100 μM pamidronate and all cells were subsequently loaded to poly-L-lysine-coated coverslips. Attached cells were fixed, permeabilized and stained with a rabbit polyclonal anti-RhoB antibody (AbCam) followed by a secondary Goat anti-Rabbit IgG AlexaFluor488-conjugated antibody (Jackson ImmunoResearch). Cells were also stained with DAPI for nuclear staining. Intracellular RhoB distribution was visualized by confocal microscopy. RhoB signal ratios between intra-nuclear and extra-nuclear compartments were quantified using ImageJ software as described previously [12].

### Animal models

NOD.Cg-PrkdcscidIl2rgtm1Wjl/SzJ (NSG) and NOD.Cg-PrkdcscidIl2rgtm1WjlTg(CMVIL3,CSF2,KITLG) 1Eav/MloySzJ (NSG-SGM3) mice originally obtained from Jackson Laboratory (Bar Harbor, ME, USA) were bred and housed in the specific pathogen-free (SPF) breeding unit of the Central Animal Facility of Utrecht University. Experiments were conducted according to Institutional Guidelines under acquired permission from the local Ethical Committee and per current Dutch laws on Animal Experimentation. Mice were housed in sterile conditions using an individually ventilated cage (IVC) system and fed with sterile food and water. Irradiated mice were given sterile water with antibiotic ciproxin for the duration of the experiment. Mice were randomized with equal distribution by sex and divided into 5 mice/group (for efficacy study) or 10 mice/group (for safety study).

Adult mice (10–14 weeks old) received sublethal total body irradiation (1.75 Gy) on Day 0. On Day 1, NSG mice were injected intravenously with 5 × 10^6^ CD3-depleted primary AML blast from donor p25 (efficacy study as PD-X model) or 0.25 × 10^6^ healthy human CD34^+^ cells from six different donors (safety study as HD-X model). Engraftment and tumor burden were followed up in the peripheral blood as described in the subsection below. When the arbitrary threshold of 500 cells/ml was reached, treatment was initiated. Mice received 2 injections of 10^7^ therapeutic TEG001 cells or TEG-LM1 mock cells (non-functional γδTCR-transduced T cells that carries length mutation of on the complementary determining region 3 (CDR3) region of the δ2-chain [18]). For second PD-X model for efficacy study, adult NSG-SGM3 mice received 2 injections of 10^7^ therapeutic TEG001 cells or TEG-LM1 mock cells at Day 8 and 16. All mice received 0.6 × 10^6^ IU of IL-2 (Proleukin; Novartis) in IFA subcutaneously together with the first T cell injection and every 21 days until the end of the experiment. Pamidronate (10 mg/kg body weight) was injected intravenously together with the first T cell injection, and every 21 days until the end of the experiment. Pamidronate was added in all our in vivo experiment in order to enhance TEGs activation as previously reported [10]. Mice were routinely monitored at least twice a week for weight loss and symptoms of disease (sign of paralysis, weakness, and reduced motility).

### Cytology staining and analysis

Cytopathologic evaluation of mouse bone marrow cytospin was performed by May-Grünwald Giemsa staining. Each sample was qualitatively and semi-quantitatively evaluated based on the following criteria: 1) cellularity (1 = high; 2 = moderate; 3 = low); 2) presence of megakaryocytes; 3) presence of all cell lineages; 4) presence of all stages of maturation for each cell lineage; 5) description of the cell types present for each cell lineage.

### Histology staining and analysis

Histopathologic evaluation was performed by hematoxylin and eosin (H&E) staining for the following mouse tissues: liver, spleen, small (duodenum, jejunum, ileum) intestine. Each organ was semi-quantitatively evaluated based on the following criteria: 1) histologic lesions were semi-quantitatively assessed (grade: 0 = absent; 1 = minimal; 2 = mild; 3 = moderate; 4 = marked); 2) the inflammation was evaluated considering the distribution (focal, multifocal, multifocal to coalescing, diffuse), severity (grade 1–4) and cell type (lymphocytes, plasma cells, macrophages, neutrophils); 3) the presence of leukemic cell infiltrate.

### Statistical analysis

Data were analyzed using GraphPad Prism (GraphPad Software Inc.) and represented as mean ± standard deviation (SD) or standard error of mean (SEM) with * *P* < 0.05; ** *P* < 0.01; and *** *P* < 0.001. Differences between groups were assessed using a two-way ANOVA, non-parametric 2-tailed Mann-Whitney t-test or Kruskal-Wallis test and Dunn’s multiple comparison test where indicated.

Cell lines, primary human materials, retroviral transduction and depletion of non-engineered T cells, CFU assays, flow cytometry analysis, assessment for human cell engraftment and preparation of single cell suspensions are described in Additional file 1.

## Results

### In vitro and in vivo activity of TEG001 against primary AML

**Fig. 1:**
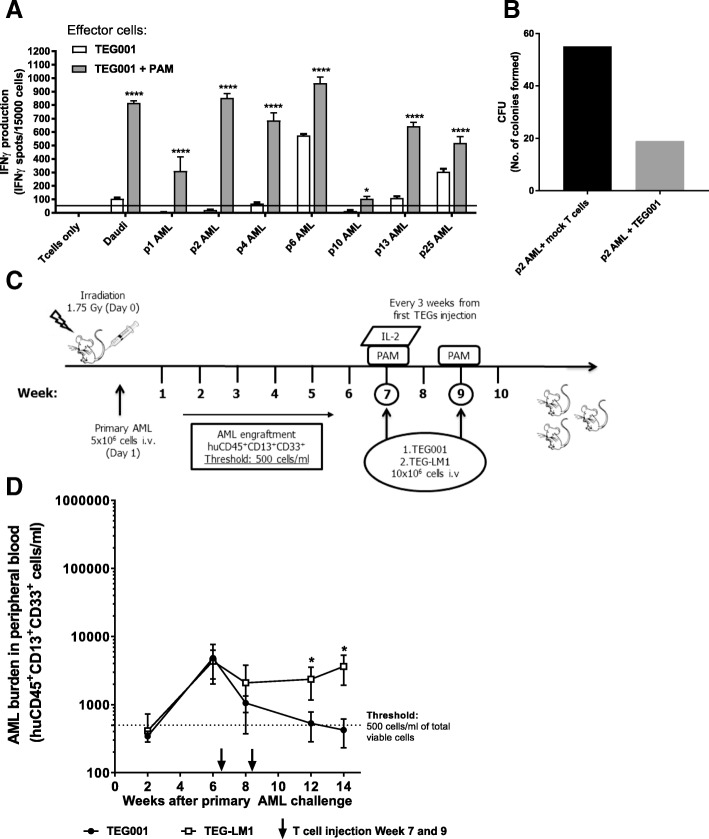
In vitro and in vivo efficacy profile of TEG001. (**a**) Antitumor reactivity of TEG001 towards patient-derived primary AML blasts in vitro. Effector cells TEG001 and primary AML blasts from multiple donors (E:T ratio is 1:3) were incubated for 24 h with or without 10 μM PAM as indicated. Daudi and healthy T cells were included as positive and negative target controls, respectively. IFNγ secretion was measured by ELISPOT. IFNγ spots per 15,000 T cells are shown as mean ± SD of at least 3 independent replicates for each target. Fifty spots/15,000 cells were considered as a positive antitumor response and indicated by the black horizontal line. Statistical significances were calculated by two-way ANOVA; *, *P* < 0.05; **, *P* < 0.01; ***, *P* < 0.001; ****, *P* < 0.0001; (**b**) Bulk αβT cells (as mock T cells) or TEG001 cells were incubated with primary AML blast from donor p2 for 5 h at E:T ratio 10:1. Then cells were plated in methylcellulose-based medium and, after 8 days, colony formation was quantified using an inverted microscope. Shown is the number of CFU formed. Data is the result of a single experiment from single primary AML donor; (**c**) Schematic overview of in vivo experiment. NSG mice were irradiated at day 0 and engrafted with primary AML cells at day 1. AML cells were followed-up in the peripheral blood by flow cytometry. When the average AML cells were > 500 cells/ml, treatment was initiated. Mice received 2 injections of therapeutic TEG001 or TEG-LM1 mock in the presence of PAM (at week 7 and 9) and IL-2 (at week 7); (**d**) Tumor burden for primary AML was measured in peripheral blood by quantifying for absolute cell number by flow cytometry. Data represent mean ± SD of all mice per group (*n* = 5). Statistical significances were calculated by non-parametric 2-tailed Mann-Whitney t-test; *, P < 0.05; **, *P* < 0.01; ***, *P* < 0.001; ****, *P* < 0.0001

Approximately 50% of the primary AML blasts tested so far are susceptible to TEG001 ( [17] and unpublished observation). We first confirmed activity of TEG001 against the primary AML blasts from multiple donors (Additional file 2: Table S1) by performing an IFNγ ELIspot assay in the presence or absence of 10 μM pamidronate (PAM) while the negative control (healthy T cells) was not recognized. Aminobiphosphonate compounds, including clinically used pamidronate, further accumulate intracellular phosphoantigens level [11]. Based on our previous study [10], the application of therapeutic concentrations of PAM enhances γ9δ2TCR recognition, including TEG001. Daudi served as a positive control. Most of the primary AML blasts could induce significant IFNγ production by TEG001 in the presence of PAM (Fig. 1A). Furthermore, we tested the cytolytic activity of TEG001 against primary AML blasts from donor p2. Primary AML blasts were incubated with either bulk αβT cells (as mock control) or with TEG001 cells in the presence of PAM on the methylcellulose matrix for the colony formation assay. Colonies were counted 8 days later. TEG001 showed a superior reduction of AML blast as shown by less colony formation in comparison to mock T cells (Fig. 1B). This result aligns with our previous data in which γ9δ2TCR-transduced αβT cells inhibited colony forming unit (CFU) of primary AML blast [10].

From this screening, we selected AML blasts from patient 25 (p25) because of its initial susceptibility to TEG001, as well as its availability in sufficient numbers for further testing in mice. Next, we injected CD3-depleted primary AML blasts from p25 into irradiated mice intravenously (Fig. 1C). Engraftment and leukemia outgrowth were detected by measuring specific AML markers huCD45^+^CD13^+^CD33^+^ in peripheral blood by flow cytometry (Additional file 3: Figure S2). When 500 AML cells/ml were detected in peripheral blood, treatment was initiated. Mice received two injections of TEG001 or TEG-LM1 mock in the presence of PAM and IL-2 (for the first TEGs injection) to support TEGs activation and proliferation in vivo. TEG-LM1 carries γ9δ2TCR with length mutation of on the CDR3 of the δ2-chain, which abrogates its function [18] and therefore chosen as a suitable mock control. γδTCR expression for both TEG001 and TEG-LM1 mock is comparable, which subsequently infused into the mice (Additional file 4: Figure S1). In the peripheral blood of the TEG001-treated mice, primary AML cells were no longer detectable five weeks after TEGs infusion, but remained measurable in mock-treated mice (Fig. 1D), suggesting that in the described PD-X model TEG001 specifically eliminates primary AML blasts over time. We further addressed the influence of microenvironment to TEG001 recognition against primary AML blasts and developed a separate PD-X model using the same p25 AML in NSG-SGM3 mice that express human cytokines (i.e. IL-3, granulocyte/macrophage colony-stimulating factor (GM-CSF), and stem cell factor (SCF)) that support better engraftment of AML blast in vivo [25]. Similarly, mice received two injections of TEG001 or TEG-LM1 mock in the presence of PAM and IL-2 (for the first TEGs injection) at Day 8 and Day 16 (Additional file 5: Figure S3A). While we did not see elimination primary AML blasts over time, TEG001-treated mice consistently showed lower AML burden in comparison to mock-treated mice as measured in peripheral blood (Additional file 5: Figure S3B). This result demonstrates antitumor activity of TEG001 against primary AML blasts in vivo as shown in two independent PD-X models.

### Assessing the activity of TEG001 against healthy hematopoiesis in vitro

**Fig. 2:**
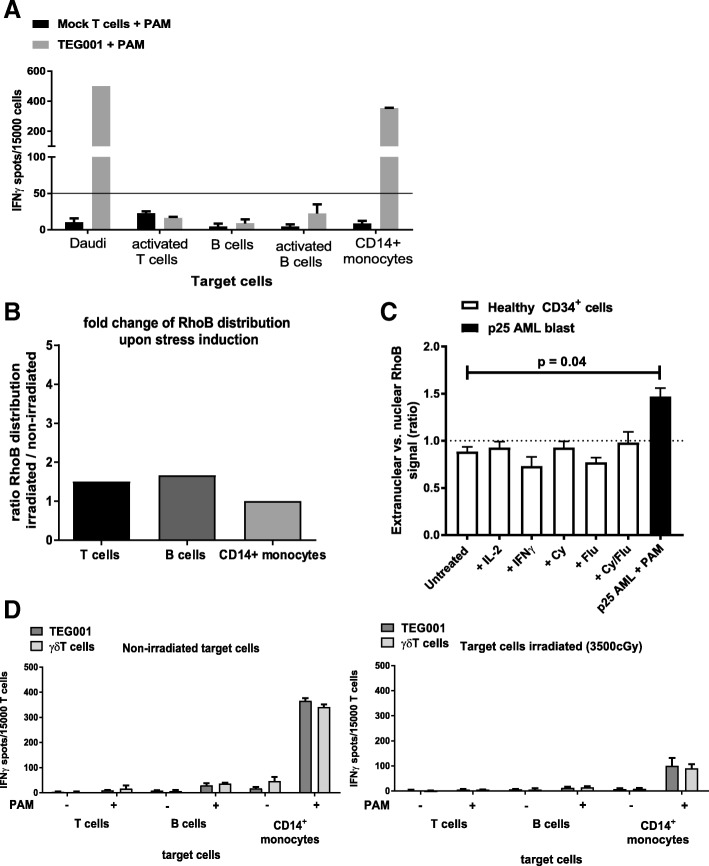
In vitro safety profile of TEG001. (**a**) Comparable recognition profile of Daudi tumor cells versus healthy hematopoietic cells. Effector cells TEG001 and target cells (E:T ratio 1:3) were incubated for 24 h in the presence of 10 μM PAM. IFNγ secretion was measured by ELISPOT. IFNγ spots per 15,000 T cells are shown as mean ± SD of at least 3 independent replicates for each target. Fifty spots/15,000 cells were considered as a positive recognition response and indicated by the black horizontal line. (**b**) RhoB distribution for healthy hematopoietic cells upon irradiation as analyzed by confocal microscopy in the presence of 10 μM PAM. Data is shown as fold-changed of RhoB distribution between irradiated cells (cellular stress condition) compared to non-irradiated cells from average ratio of at least ten different cells; (**c**) CD34^+^ progenitor cells from a healthy donor were treated with either 50 IU/ml IL-2, 1000 IU/ml IFNγ, 5 mM Cy, 20 μM Flu or combination of Cy/Flu. As positive control primary AML blast from donor p25 was treated with PAM. All cells were analyzed for intracellular distribution of RhoB using confocal microscopy. White bars represent healthy CD34^+^ progenitor cells, while black bar indicate primary AML blast (p25 AML). The RhoB signal ratio between nuclear and extranuclear cellular compartments was measured using ImageJ image analysis software. Graphs show average ratios of at least ten different cells ±SEM. Statistical significance compared to untreated CD34^+^ progenitor cells was determined by using Kruskal-Wallis test and Dunn’s multiple comparison test; (**d**) Comparable recognition profile of healthy hematopoietic cells in non-stressed (left panel) and stressed (irradiated, right panel) conditions. Effector cells TEG001 and target cells (E:T ratio 1:3) were incubated for 24 h in the presence of 100 μM PAM. IFNγ secretion was measured by ELISPOT. IFNγ spots per 15,000 T cells are shown as mean ± SD of at least 3 independent replicates for each target

Next, we aimed to assess the toxicity of TEG001 against the hematopoietic compartment in vitro. Therefore, TEG001 and mock transduced T cells were incubated with the physiological hematopoietic target of γδT cells, namely CD14^+^ monocytes, activated T cells as well as non-activated and activated B cells in the absence and presence of PAM. Similar to the efficacy study, we include the presence of PAM to enhance TEG001 recognition as previously shown [10]. Daudi served again as a positive control. In an IFNγ ELIspot assay cytokine secretion was only observed against the positive control and CD14^+^ monocytes in the presence of PAM, while other T and B cells did not induce IFNγ production (Fig. 2A).

Translocation of RhoB towards the cell membrane has been described as a key step for the recognition of a potential target by a γ9δ2TCR [12]. This insight allowed us to test whether an additional stress of hematopoietic cells would activate this key step in the mode of action and thereby facilitate recognition of healthy compartments. As “stress” we have chosen irradiation, which is well known to activate many innate danger signals like MHC-like molecules [26], and is frequently used as preconditioning before the transfer of immune cells [27]. Therefore, we assessed the translocation of RhoB towards the cell membrane in T cells, B cells and CD14^+^ monocytes in the absence and presence of irradiation. No significant increase in translocation of RhoB to the cell membrane could be observed (Fig. 2B) for the tested healthy hematopoietic cells. We also assessed the RhoB localization in healthy CD34^+^ progenitor cells upon stimulation with cytokines, such as IL-2 and IFNγ, as well as the presence of chemotherapy agents Cy/Flu and compared to primary AML blast from donor p25. While there is a significant increase in RhoB localization towards cell membrane in p25 AML blast, there are no significant increased for the healthy CD34^+^ progenitor cells in all conditions (Fig. 2C). Furthermore, the recognition profile by TEG001 of the same cell subsets after irradiation was assessed by cytokine secretion. Recognition of a priori non-recognized cells was not induced and recognition of CD14^+^ monocytes was slightly reduced after irradiation (Fig. 2D). Overall, our results suggest that TEG001 does not attack subsets of healthy hematopoiesis in the absence or presence of stress. Only CD14^+^ monocytes can be recognized in the presence of PAM as reported also for natural γ9δ2T cells [10].

### In vivo pharmacology and toxicology of TEG001

**Fig. 3:**
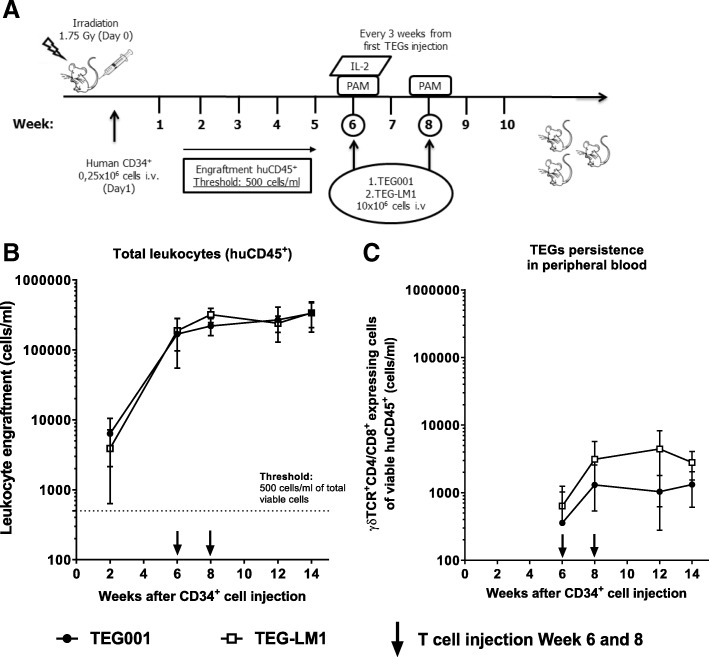
In vivo safety profile of TEG001. (**a**) Schematic overview of the safety experiment in healthy donor-derived xenograft (H-DX) model. NSG mice were irradiated at day 0 and engrafted with healthy cord blood-derived CD34^+^ progenitor cells on day 1. Engraftment was followed-up in peripheral blood by flow cytometry and when > 500 huCD45^+^ cells/ml were present, mice received 2 injections of therapeutic TEG001 or TEG-LM1 mock in the presence of PAM (at week 6 and 8) and IL-2 (at week 6); (**b**-**c**) In vivo safety profile of TEG001 towards healthy human hematopoietic cells. Healthy human cells engrafted in NSG mice (**b**) with long-term persistence of TEGs in peripheral blood (**c**). Data represent mean ± SD of all mice per group (*n* = 10)

Assessment of different hematopoietic subsets by TEGs in vitro is very restricted due to the limited sub-fractions available for testing. In addition, it does not allow for assessment of whether early precursors are affected. Therefore, we established a HD-X model with human cord-blood derived CD34^+^ progenitor cells from six healthy donors repopulated in irradiated mice to further assess the safety profile of TEG001 against the hematopoietic compartment (Fig. 3A). Engraftment of human leukocytes (huCD45^+^) and other hematopoietic cellular subsets in peripheral blood was measured by flow cytometry (Additional file 6: Figure S4A and S4B). When 500 huCD45^+^ cells/ml were detected in peripheral blood, treatment was initiated with either TEG001 or TEG-LM1 mock in the presence of PAM and IL-2 (for the first TEGs injection) to support TEGs activation and proliferation in vivo. While we observed a reduction of tumor burden by TEG001 (Fig. 1D), no significant differences in engraftment of healthy hematopoietic cells between treatment groups were observed up to 50 days after infusion when assessed by huCD45^+^ (Fig. 3B). TEG001 as well as TEG-LM1 cells could be detected after injection until the end of the study period in the peripheral blood of mice (Fig. 3C).

**Fig. 4:**
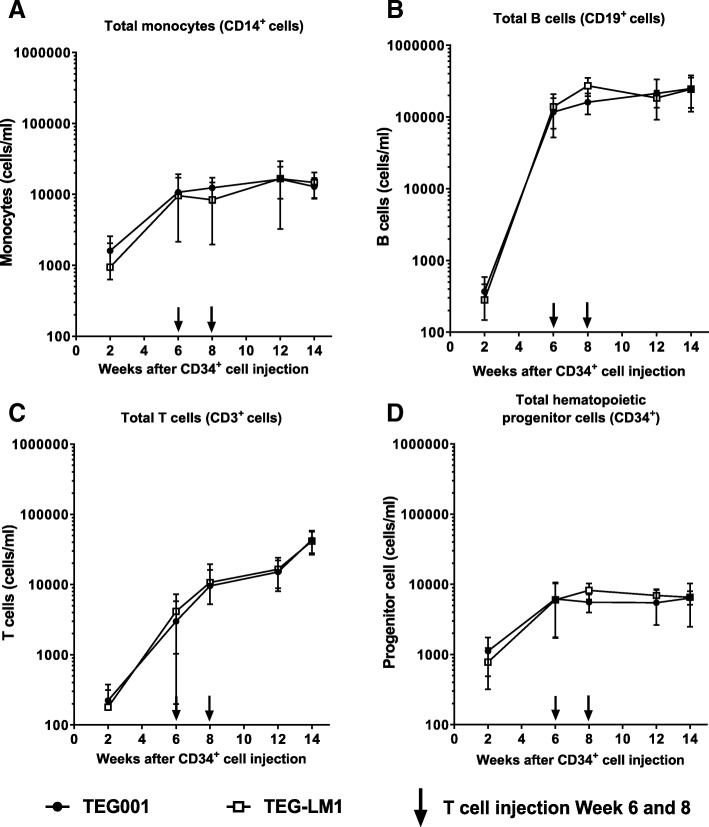
In vivo reconstitution of healthy human hematopoietic compartments in peripheral blood. Comparable profile between TEG001 and TEG-LM1 mock group of reconstituted healthy human hematopoietic cellular subsets, including CD14^+^ monocytes (**a**) B cells (**b**), T cells (**c**), and CD34^+^ progenitor cells (**d**) as measured by flow cytometry. Data represent mean ± SD of all mice per group (n = 10)

**Fig. 5:**
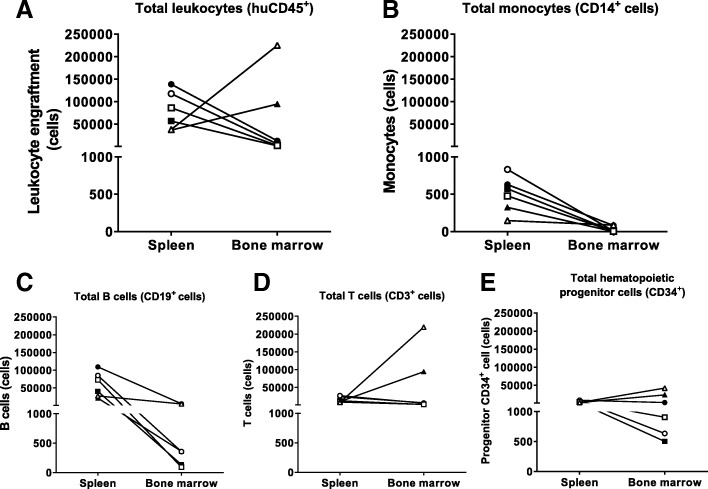
In vivo reconstitution of healthy human hematopoietic compartments in tissues. Comparable profile between TEG001 and TEG-LM1 mock group of reconstituted healthy human hematopoietic cellular subsets in spleen and bone marrow, including total human CD45^+^ leukocytes (**a**), CD14^+^ monocytes (**b**), B cells (**c**), T cells (**d**) and CD34^+^ progenitor cells (**e**) as measured by flow cytometry. Shown in the data from individual mouse (represented by different symbols) of both TEG001 (filled symbol) and TEG-LM1 mock (open symbol) group (*n* = 3 mice/group)

Next, we investigated the reconstitution of diverse hematopoietic cellular subsets in vivo in more detail in the peripheral blood of mice. In particular, we were interested in the impact on monocytes given that in vitro natural γ9δ2T cells as well as TEG001 can recognize primary monocytes. Interestingly, neither CD14^+^ monocytes, nor CD19^+^ B cells, CD3^+^ T cells, or CD34^+^ progenitor cells were impaired in outgrowth when comparing mice injected with TEG001 or TEG-LM1 (Fig. 4A-D). At the end of study period, we also obtained single cell suspension from spleen and bone marrow from three mice for both the TEG001 and TEG-LM1 mock group to analyze the reconstitution of similar cell subsets in more detail in primary tissues (Fig. 5A-E). In line with our observations in peripheral blood, we could observe all relevant subsets, namely CD14^+^ monocytes, CD19^+^ B cells, CD3^+^ T cells, and CD34^+^ progenitor cells.

**Fig. 6:**
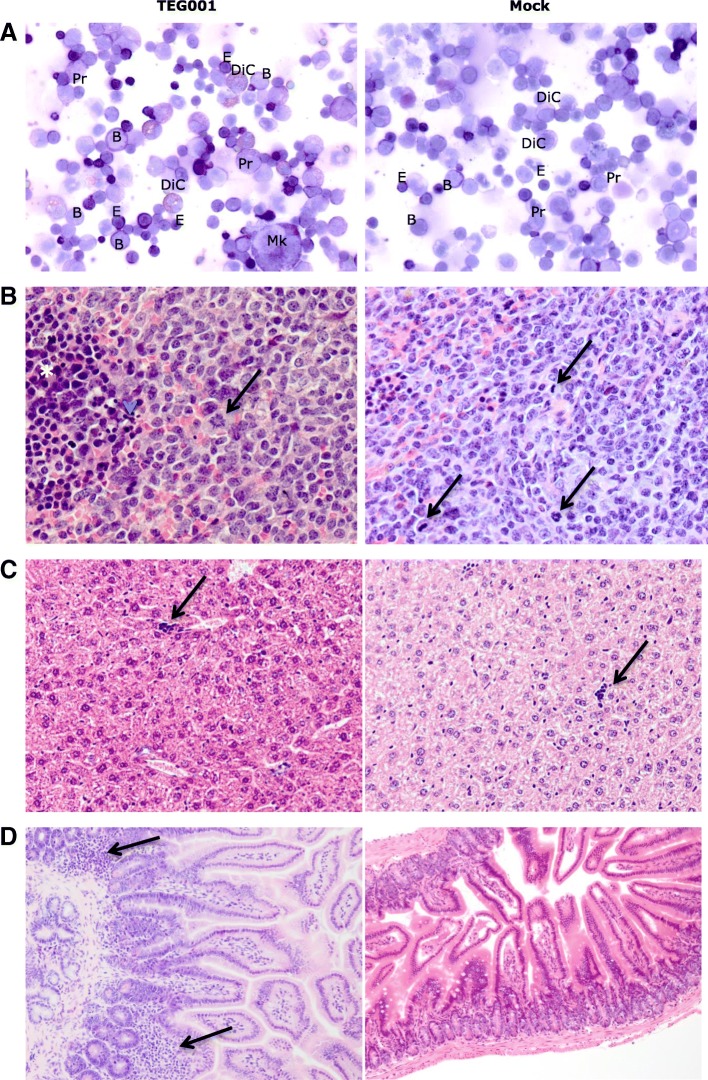
Cytopathology analysis of bone marrow and histopathology analysis of mouse vital organs (spleen, liver, intestine). (**a**) Representative picture of May-Grünwald Giemsa staining for bone marrow cytospin from both treatment groups (TEG001 and TEG-LM1 mock) with pleomorphic population of cells with all maturation stages including numerous blasts (B), promyelocytes (Pr), dysplastic immature cells (DiC), megakaryocytes (Mk) and a mixed population of myeloid and erythroid (E) lineages; (**b**) Representative pictures for H&E staining of mouse spleen for both treatment group (TEG001 and TEG-LM1 mock) with non-neoplastic, lympho/histiocytic hyperplastic lesion with mitotic figure (arrows), apoptotic bodies (arrowhead) and erythroid precursors (*). Magnification: 40X; (**c**) Representative pictures for H&E staining of mouse liver for both treatment group (TEG001 and TEG-LM1 mock) with small focus of extramedullary hematopoiesis (arrows) in all samples, which could be due to the mouse model with engraftment of human CD34^+^ progenitor cells. Magnification: 20X; (**d**) Representative pictures for H&E staining of mouse intestine for TEG001-treated group (left) showing multifocal lymphocytic infiltration of lymphoid cells (arrows) in a small tract of the small intestine (background lesion) and TEG-LM1 mock-treated group (right) with normal jejunum. Magnification: 10X. Shown are representative pictures from an individual mouse of both TEG001 and TEG-LM1 mock group (n = 3 mice/group) with no significant differences in overall cytopathology and histology features between treatment groups

We next collected bone marrow cytospin samples from the same mice for a more detailed cytopathology analyses. All of the bone marrow samples from both treatment groups showed a pleomorphic population of cells derived from erythroid and myeloid lineages, with all the maturation stages (Fig. 6A). In almost all samples (5/6) eosinophilic differentiation was also evident. Beside normal blasts, an immature population with altered morphology (dysplastic immune cells), consistent with granular blasts was detected in both TEG001 and TEG-LM1 treated mice as well as cells with blast-like phenotypes with an indented nucleous, consistent with promonocytes (4/6), but no leukemic features were observed. Importantly, we did not observe any apparent differences in their outgrowth between treatment groups indicating that TEG001 do not harm the reconstitution of healthy hematopoietic compartments in vivo.

To evaluate off-target toxicity of TEG001 towards healthy tissues not related to the known mode of action which is absent in mice [21], we collected further mouse spleen, liver and intestine from three mice for each TEG001 and TEG-LM1 mock group and performed histopathology analyses. Spleen tissues showed non-neoplastic, lympho/histiocytic proliferative lesions in all the examined samples, of both treatment groups (Fig. 6B). Similarly, no significant histological features of toxicity or other relevant abnormalities were observed in liver or intestine in all samples (Fig. 6C-D). Most of the samples showed extramedullary hematopoiesis, mainly involving the erythroid lineage (extramedullary erythropoiesis) with scattered megakaryocytes sometimes evident, as a possible consequence of the engrafted human CD34^+^ progenitor cells in this mouse model. Overall, our data indicate there are no significant differences in histology features and notably, there are no off-target toxicities observed in all healthy tissues upon TEG001 treatment.

## Discussion

TEG001 has been selected as the first candidate for clinical testing (NTR6541) based on its superior recognition of hematological malignancies against both cell lines and primary AML in vitro and its ability to limit the tumor outgrowth in cell line-derived xenograft mouse models [10, 17, 18]. Within this study, we have been able, for the very first time, to assess therapeutic efficacy towards primary AML blasts in a clinically relevant model with established leukemic load in vivo*,* while excluding toxicity against other hematopoietic stem cell compartments. Our current observation that primary AML can be eliminated in an in vivo model by TEG001, without affecting the hematopoietic compartment, is in line with our previous observation that an alteration in the RhoB-CD277J axis, the putative ligand of γ9δ2TCR, is selectively observed in the leukemic but not healthy hematopoietic stem cell [12].

A major challenge a priori clinical testing of novel cell-based and gene therapy products remains to assess efficacy and toxicity in relevant pre-clinical models in order to avoid unwanted toxicities like those reported for different CAR-T [28] or αβTCR gene therapy programs [29]. This reflects the quite different characteristics of cell-based gene therapy medicinal products in comparison to conventional synthetic drugs. Thus, classical clinical considerations of therapeutic efficacy and safety assessments might no longer apply for these ‘living’ medicinal products. With TEG001, a next level of complexity is introduced due to the nature of the target. In contrast to, e.g., CD19-directed CAR T gene therapy, which targets a very well-defined protein expressed on cancer cells and B cells [5], TEG001 is targeting metabolic changes in stressed and malignant cells, driven by a dysregulated mevalonate pathway [11]. Although transfer of conventional γ9δ2T cells has not been reported to associate with substantial toxicity [13], the TEG concepts express an activating γ9δ2TCR outside the context of its natural brakes, through a plethora of killer immunoglobulin-like receptor (KIR) inhibitory receptors usually operational in natural γ9δ2T cells. Therefore, Dutch authorities have required additional safety tests for TEG001 prior to clinical testing. However, dysregulated metabolic pathways do not allow for high throughput evaluations of the ligand in all tissues through, e.g., gene expression or transcriptome analyses [30]. Consequently, following the advice of the Dutch authorities, our group developed different strategies to test the efficacy and safety of TEG001 in models where healthy and malignant cells are present either simultaneously or sequentially. One such model is a 3D bone marrow model where primary multiple myeloma cells grow out along with healthy stromal cells into an artificial bone marrow niche. Upon TEG001 injection, this model confirmed the activity of TEG001 against the malignant fraction, but not healthy bystander cells present in the bone marrow niche [24]. However, the 3D bone marrow niche is also limited, as it does not allow for engrafting of the complex hematopoietic system and or assessing toxicity against all cellular compartments usually generated from a hematopoietic stem cell.

To study the interaction between tumor and immune cells, we have to consider also their interaction within the same microenvironment. Xia and colleagues [31] develop humanized mice model with human hematopoietic system and autologous leukemia in the same individual mouse. This model is developed by transducing CD34^+^ fetal liver cells with retroviral vector containing mixed-lineage leukemia MLL-AF9 fusion gene, which allows recapitulation of human leukemic diseases [31, 32]. Although it would be interesting to develop a similar humanized mouse model in which healthy human hematopoietic cells and primary leukemic blasts presence in the same individual mouse, the availability of healthy human CD34^+^ progenitor cells from the very same leukemia patient is a limiting factor. Hence, we develop two separate mice models and thereby avoiding limiting criteria of HLA-matching between healthy CD34^+^ progenitor cells and primary AML donors.

In order to test the efficacy of TEG001, we utilized a mouse xenograft model, which has been widely used to study therapeutic responses in heterogeneous diseases such as cancer. PD-X models, considered to closely mimic human diseases, are established by engrafting primary patient material into immunodeficient mice [33]. Assessment of AML burden in mouse xenograft models is commonly performed by measuring the percentage of human leukemic cells in bone marrow at the end of study period. In this study, we developed a stringent treatment model where we infused TEG001 upon the onset of the disease (represented by an arbitrary threshold of 500 AML cells/ml detected in peripheral blood). Moreover, we developed an elegant method that allowed us to follow the disease progression for a longer period as well as the treatment effect to reduce tumor burden over time by measuring AML cells in peripheral blood. Nonetheless, we acknowledged some limitations in our method, such as variable engraftment rates commonly observed in PD-X model [34] and a low level of AML engraftment in peripheral blood of adult NSG mice as reported previously [35]. In spite of these limitations, we were able to detect a significant reduction of AML cells in peripheral blood of TEG001-treated mice in comparison to the mock-treated group. Furthermore, we developed a separate PD-X model using NSG-SGM3 mice using the same primary AML blast from donor p25 to assess the influence of microenvironment towards TEG001 efficacy profile. NSG-SGM3 mice express human cytokines, including IL-3, GM-SCF, and SCF, and thereby supporting primary AML engraftment and their survival in vivo [25]. Here we demonstrate that TEG001-treated group showed significantly lower AML burden in comparison to mock-treated group, despite the lack of tumor clearance. This could be due to the more protective microenvironment poses by NSG-SGM3 mice, which could hamper T cell access to target cells and therefore limit the ability of TEG001 to clear primary AML burden over time. Based on the overall data and thus as proof-of-principle we have demonstrated the efficacy profile of TEG001 against primary human AML in two independent models.

In order to assess the toxicity of TEG001 against the hematopoietic compartment in the very same model we engrafted NSG mice with CD34^+^ progenitor cells derived from healthy human cord blood donors. Reconstitution of hematopoietic cellular compartments when assessed in the peripheral blood occurred at different stages, in which CD14^+^ monocytes and CD19^+^ B cells significantly increased two weeks after progenitor cell injection, whereas CD3^+^ T cells reconstituted relatively slower, however no differences could be observed between TEG001 and mock-treated mice. Furthermore, we investigated whether TEG001 does affect hematopoietic compartments in different tissues, specifically spleen and bone marrow, at which progenitor cells should reside [36, 37]. While we could find all equivalent cell subsets with comparable reconstitution for both treatment groups also in spleen and bone marrow, there were differences in the prevalence for CD14^+^ monocytes and CD19^+^ B cells in different tissues, however again with no difference between TEG001 and mock treated mice. Monocytes were found in higher numbers in the peripheral blood when compared to bone marrow and spleen, whereas B cells were prevalently observed in the periphery and spleen. This observation is in line with previous studies showing that the reconstitution of human hematopoietic stem cells in host mice is commences predominantly with erythroid and myeloid cells, followed by lymphoid progenitor and lastly mature lymphocytes [38]. Also, neither induction of cellular stress by irradiation nor exposure to inflammatory cytokines (i.e., IL-2 and IFNγ), or the presence of chemotherapy agent Cy/Flu alter RhoB translocation towards the cell membrane for healthy CD34^+^ progenitor cells, and thus no alteration of TEG001 recognition pattern. In addition, our data confirm that different tissue compartments are comprised of different types of immune cells; and show that TEG001 treatment did not influence this pattern. Thus TEG001 most likely does not affect homing of hematological subsets nor mediate hematopoietic toxicity, as suggested by our previous work demonstrating that the mode of action is mainly observed in tumor cells and not in the healthy hematopoietic compartment [12, 19]. The only physiological target of γ9δ2TCRs are professional antigen presenting cells (APC) like monocytes and dendritic cells in the presence of PAM [18], as also demonstrated in this study in the in vitro experiments. However, as reported previously, this recognition apparently fosters the maturation of APC and potentially broadens an adaptive immune response through epitope spreading [39] rather than promoting elimination of APC. In line with this assumption, we could still detect CD14^+^ monocytes reconstitution in vivo after transfer of TEG001.

## Conclusion

In conclusion, our data suggest antitumor reactivity of TEG001 against primary AML blasts in vivo. While we concur that the absence of evidence is not equal to the evidence of absence and within the limitation of our current models where off-target activities cannot be excluded entirely, there are no data indicating an increased safety risk specific for TEG001. A GMP-compliant production of TEG001 has now been established [17, 40], and will be used in an ongoing phase I open-label dose escalation study to explore toxicity and activity of TEG001 in patients with primary refractory or relapsed acute myeloid leukemia, as well as patients with multiple myeloma.

## Additional files

Additional file 1: Supplementary Materials and methods including cell lines, primary materials, retroviral transduction and depletion of non-engineered T cells, CFU assay, flow cytometry analysis, assessment for human cell engraftment and Preparation of single cell suspensions. (DOCX 25 kb)

Additional file 2: **Table S1.** Primary AML characteristic. Characteristic of primary AML materials from different donors. (DOCX 17 kb)

Additional file 3: **Figure S2.** Gating strategy for flow cytometry analysis of primary AML burden. A representative flow cytometry plot of murine peripheral blood. Tumor load was measured by quantifying absolute cell number of viable huCD45^+^CD13^+^CD33^+^ of the primary AML blast and representative plot for TEG001 and TEG-LM1 mock group. (PPTX 178 kb)

Additional file 4: **Figure S1.** γδTCR expression of TEG001 and TEG-LM1 mock. A representative flow cytometry plot γδTCR expression of TEG001 and TEG-LM1 mock after transductions, after αβTCR depletion and prior to infusion into mice after 2 weeks expansion. (PPTX 191 kb)

Additional file 5: **Figure S3.** In vivo efficacy profile of TEG001 in PD-X model of primary blast in NSG-SGM3 mice. (A) Schematic overview of in vivo experiment. NSG-SGM3 mice were irradiated at day 0 and engrafted with primary AML cells at day 1. AML cells were followed-up in the peripheral blood by flow cytometry. Mice received 2 injections of therapeutic TEG001 or TEG-LM1 mock in the presence of PAM (at Day 8 and 16) and IL-2 (at Day 8); (B) Tumor burden for primary AML was measured in peripheral blood by quantifying for absolute cell number by flow cytometry. Data represent mean ± SD of all mice per group (*n* = 5 mice/group). Statistical significances were calculated by non-parametric 2-tailed Mann-Whitney t-test; *, *P* < 0.05; **, *P* < 0.01; ***, *P* < 0.001; ****, *P* < 0.0001. (PPTX 101 kb)

Additional file 6: **Figure S4.** Gating strategy for flow cytometry analysis of healthy hematopoietic compartments. A representative flow cytometry plot of murine peripheral blood. (A) Engraftment was determined by quantifying absolute cell number of viable huCD45^+^ of healthy stem cells; (B) Hematopoietic cellular compartments outgrowth were determined by quantifying absolute cell number for CD19^+^ B cells, CD3^+^ T cells, and CD14^+^ monocytes. Also, persistence of TEGs were determined by quantifying absolute cell number for γδTCR^+^ cells. (PPTX 225 kb)

## Abbreviations

AML: Acute myeloid leukemia
APC: Antigen presenting cells
CAR-T: Chimeric antigen receptor T cells
CDR: Complementary Determining Region
CFU: Colony Forming Unit
Cy: Cyclophosphamide
FCS: Fetal calf serum
Flu: Fludarabine-phosphate
GMP: Good Manufacturing Practice
GM-SCF: Granulocyte/Macrophage Colony-Stimulating Factor
H&E: Hematoxylin and eosin
HD-X: Healthy donor-derived xenograft
IVC: Individually ventilated cage
KIR: Killer immunoglobulin-like receptor
NKG2D: Natural-killer group 2, member D
PAM: Pamidronate
PBMCs: Peripheral blood mononuclear cells
PD-X: Patient-derived xenograft
SCF: Stem Cell Factor
SD: Standard deviation
SEM: Standard error of mean
SPF: Specific pathogen-free
TCR: T cell receptor
TEGs: αβT cells Engineered to express a defined γδTCR
