# Supplementary material for: Evaluating in vivo efficacy – toxicity profile of TEG001 in humanized mice xenografts against primary human AML disease and healthy hematopoietic cells
Source: J Immunother Cancer. 2019 Mar 12;7:69. doi: 10.1186/s40425-019-0558-4 (PMC6419469; doi:10.1186/s40425-019-0558-4)
Supplement: Supplementary file 1 — Supplementary Materials and methods including cell lines, primary materials, retroviral transduction and depletion of non-engineered T cells, CFU assay, flow cytometry analysis, assessment for human cell engraftment and Preparation of single cell suspensions. (DOCX 25 kb) [file 40425_2019_558_MOESM1_ESM.docx]

**Supplementary materials and methods**

**Cells and cell lines**

Daudi and Phoenix-Ampho were obtained from ATCC (authenticated by short tandem repeat profiling/karyotyping/isoenzyme analysis). Daudi cells were cultured in RPMI media supplemented with 10% fetal calf serum (FCS) and 1% Pen/Strep. Phoenix-Ampho cells were cultured in DMEM media supplemented with 10% FCS and 1% Pen/Strep. All cells were passaged for a maximum of 2 months, after which new seed stocks were thawed for experimental use. Furthermore, all cell lines were routinely verified by growth rate, morphology, and/or flow cytometry and tested negative for mycoplasma using MycoAlert Mycoplasma Kit. Peripheral blood mononuclear cells (PBMCs) were isolated using Ficoll gradient centrifugation methods from buffy coats obtained from Sanquin Blood bank (Amsterdam, The Netherlands).

**Primary materials**

Primary AML blasts were obtained from biobank of University Medical Center Utrecht in accordance with good clinical practice and Declaration of Helsinki regulations. All patients gave their consent prior to storage in the biobank (TCBio 16-088). For *in vivo* experiments, apheresis material from primary AML material from donor p25 was depleted for CD3^+^ cells using human CD3 Microbeads (Miltenyi Biotech) per the manufacturer’s protocol. Human CD34^+^ derived from cord blood of six healthy donors were isolated using anti-CD34 magnetic beads separation (Miltenyi Biotec), and are obtained as a kind gift from Dr. Maud Platinga (University Medical Center Utrecht).

**Retroviral transductions of T cells**

TEGs were produced as previously described (10). Briefly, packaging cells (Phoenix-Ampho) were transfected with helper constructs gag-pol (pHIT60), env (pCOLT-GALV) and pMP71 retroviral vectors containing both γδTCR chains separated by a ribosomal skipping T2A sequence, using FugeneHD reagent (Promega). Human PBMCs from a healthy donor were pre-activated with anti‑CD3 (30 ng/mL; Orthoclone OKT3; Janssen-Cilag) and IL-2 (50 IU/mL; Proleukin, Novartis) and subsequently transduced twice with viral supernatant within 48 hours in the presence of 50 IU/mL IL-2 and 6 mg/mL polybrene (Sigma-Aldrich). TCR-transduced T cells were expanded by stimulation with anti-CD3/CD28 Dynabeads (500,000 beads/10^6^ cells; Life Technologies) and IL-2 (50 IU/mL). Thereafter, TCR-transduced T cells were depleted of the non-engineered T cells.

**Depletion of non-engineered T cells**

Depletion of non-engineered T cells was performed as previously described (17). Briefly, γδTCRs transduced αβT cells were incubated with a biotin-labeled anti-αβTCR antibody (clone BW242/412; Miltenyi Biotec) and subsequently incubated with an anti-biotin antibody coupled to magnetic beads (anti-biotin MicroBeads; Miltenyi Biotec). Thereafter, the cell suspension was loaded onto an LD column and αβTCR^+^ T cells were depleted by MACS cell separation per the manufacturer’s protocol (Miltenyi Biotec). After depletion, TEGs were expanded using T cell REP.

**CFU assay**

Colony formation unit assay: 0.125 × 10^6^ primary AML blasts from donor p2 were pre-incubated with medium only with 1.25 × 10^6^ TEG001 or bulk αβT cells (as mock T cells) in the presence of 100 µM pamidronate (PAM) for 5 hours at 37°C prior to plating in Methylcellulose-based medium with recombinant cytokines (MethoCult™ H4434 Classic, StemCell Technologies). Cultures were incubated in 37°at 5% CO_2_ for 8 days and colonies were counted using an inverted microscope.

**Flow cytometry analysis**

The following antibodies were used for flow cytometry analysis: huCD45-PB (clone HI30, Sony Biotechnology), mCD45-APC (clone 30-F11, Sony Biotechnology), CD13-PECy7 (clonw WM15, Sony Biotechnology), CD33-BV711 (clone WM33, Sony Biotechnology), pan-γδTCR-PE (clone IMMU510, Beckman-Coulter), CD3-AF700 (clone UCHT1, Biolegend), CD8-PerCPCy5.5 (clone RPA-T8, Biolegend), CD8-FITC (clone TPA-T8, BD Biosciences), CD4-FITC (clone TPA-R4, Biolegend), CD34-BV650 (clone 581, BD Biosciences), CD19-PerCPCy5.5 (clone HIB19, BD Biosciences), CD14-APCeFluor780 (clone 61D3, eBioscience). To exclude non-viable cells from the analysis, Fixable Viability Dye eFluor506 was used (eBioscience). All samples were analyzed on BD LSRFortessa using FACSDiva Software (BD Biosciences).

**Assessment for human cell engraftment**

Peripheral blood samples were obtained via cheek vein (max. 100-200µl/mouse) every 1-2 weeks. Human cells in peripheral blood were quantified using Flow-count Fluorospheres (Beckman Coulter). Red blood cell lysis was performed for blood samples using 1X RBC lysis buffer (Biolegend) before cell staining. Blood samples were stained with a mixed of antibody panels as listed above. Engraftment and tumor burden was measured in peripheral blood by quantifying for absolute cell number by flow cytometry using specific markers huCD45^+^CD13^+^CD33^+^ for primary AML blasts and huCD45^+^ for healthy progenitor cells, respectively. An arbitrary threshold of 500 cells/ml was chosen to represent established human cell engraftment.

**Preparation of single cell suspensions**

At the end of the study, bone marrow (mixed from tibia and femur) and spleen sections were isolated and processed into single cell suspension. Femur and tibia from the hind legs were collected; bone marrow cells were collected by centrifugation of the bones at 10,000 rpm for 15 seconds and resuspension of the cells in RPMI media. Bone marrow cells were also used to make cytospin slides (1 million cells per slide per mouse) for further cytopathology analysis.

A small section of the spleen was minced and passed through a 70µm cell strainer (BD); cells were washed in PBS and resuspended in RPMI media. A total of 500,000 cells were stained and analyzed for human hematopoietic cellular compartments by flow cytometry analysis (BD LSRFortessa). Human cells from spleen and bone marrow were measured by quantifying absolute cell number from total 500,000 cells using Flow-count Fluorospheres (Beckman Coulter) for each hematopoietic cellular compartment.
