# Supplementary material for: Evaluating in vivo efficacy – toxicity profile of TEG001 in humanized mice xenografts against primary human AML disease and healthy hematopoietic cells
Source: J Immunother Cancer. 2019 Mar 12;7:69. doi: 10.1186/s40425-019-0558-4 (PMC6419469; doi:10.1186/s40425-019-0558-4)
Supplement: Supplementary file 2 — Table S1. Primary AML characteristic. Characteristic of primary AML materials from different donors. (DOCX 17 kb) [file 40425_2019_558_MOESM2_ESM.docx]

**Table S1.** Characterization of primary AML-patient samples

| **Donor** | **Classification ^1^** | **% blast ^2^** | **Major phenotype** | **Cytogenetic** | **Risk classification** |
| --- | --- | --- | --- | --- | --- |
| **p1** | Transformation of CML to myeloid blast crisis | 80% | CD34^+^, CD117^+^, CD13^+^, CD36^+^, CD33^+^, CD45^low^, HLA-DR^+^, MPO^+^, CD19^+^, CD22^+^, cTdT^+^, CD79a^+^ | BCR-ABL1 fusion  mRNA type Major (~t(9;22)(q34;q1)) | N/A ^3^ |
| **p2** | M5 | 47% blasts,  18% monocytic cells | Blasts: CD34^+^, CD117^+^, HDL-DR^+^, CD13^+^, CD7^+^, CD11c^+^, CD56^+^, cCD3^low^, cMPO^low^.  Monocytic cells: CD36^+^, CD14^+^, CD11b^+^, CD13^+^, CD33^+^, HLA-DR^+^, CD3^low^, CD56^+^ | Monosomy chromosome 7 | Poor |
| **p4** | M4eo | 46% | CD34^+^, CD117^+^, CD45^low^, CD33^+^, CD13^+^, HLA-DR^+^, CD38^low^, CD65^+^, cMPO^+^, cTDT^low^ | Inversion chromosome 16  (inv(16)(p13q22)) | Good |
| **p6** | AML with monocytic maturation | 88% | CD45^low^, CD33^+^, CD11b^low^, CD11c^+^, CD15^+^, CD36^low^, CD65^+^, CD4^low^, CD117^+^, HLA-DR^+^, CD38^+^, CD56^+^, CD113^+^, CD79a^+^ | Complex karyotype | Very poor |
| **p10** | M4/M5 with extramedullary manifestations | 50% | CD45^low^, CD117^low^, cMPO^low^, CD13^+^, CD33^+^, CD38^+^, HLA-DR^+^, CD11b^low^, CD36^+^, CD65^+^, CD4^low^ | Normal karyotype  No abnormalities detected | Poor |
| **p13** | M5 | 85% | CD45^low^, CD33^+^, CD38^+^, CD4^low^, CD11c^+^, cCD79a^+^, cMPO^+^, CD14^+^, CD13^+^, CD36^+^, HLA-DR^+^, CD11b^+^, CD65^+^ | MLL-MLLT3 fusion mRNA, 46XY, (t(9;11)(p21;q23)) | Poor |
| **p25** | M5A | 96% | CD45^low^, cMPO^+^, CD34^+^, CD117^+^, CD33^+^, CD13^+^, CD38^+^, HLA‑DR^+^, CD36^+^, CD14^+^, CD15^+^ and CD11b^+^ | Extra chromosome 8  No other abnormalities | Poor |

^1^ Based on the French-American-British (FAB) classification in accordance to WHO 2008

^2^ Measured in the blood at the moment of diagnosis

^3^ Not applicable, data is not available for this patient
