# Supplementary material for: Evaluating in vivo efficacy – toxicity profile of TEG001 in humanized mice xenografts against primary human AML disease and healthy hematopoietic cells
Source: J Immunother Cancer. 2019 Mar 12;7:69. doi: 10.1186/s40425-019-0558-4 (PMC6419469; doi:10.1186/s40425-019-0558-4)
Supplement: Supplementary file 3 — Figure S2. Gating strategy for flow cytometry analysis of primary AML burden. A representative flow cytometry plot of murine peripheral blood. Tumor load was measured by quantifying absolute cell number of viable huCD45+CD13+CD33+ of the primary AML blast and representative plot for TEG001 and TEG-LM1 mock group. (PPTX 178 kb) [file 40425_2019_558_MOESM3_ESM.pptx]

## Slide 1
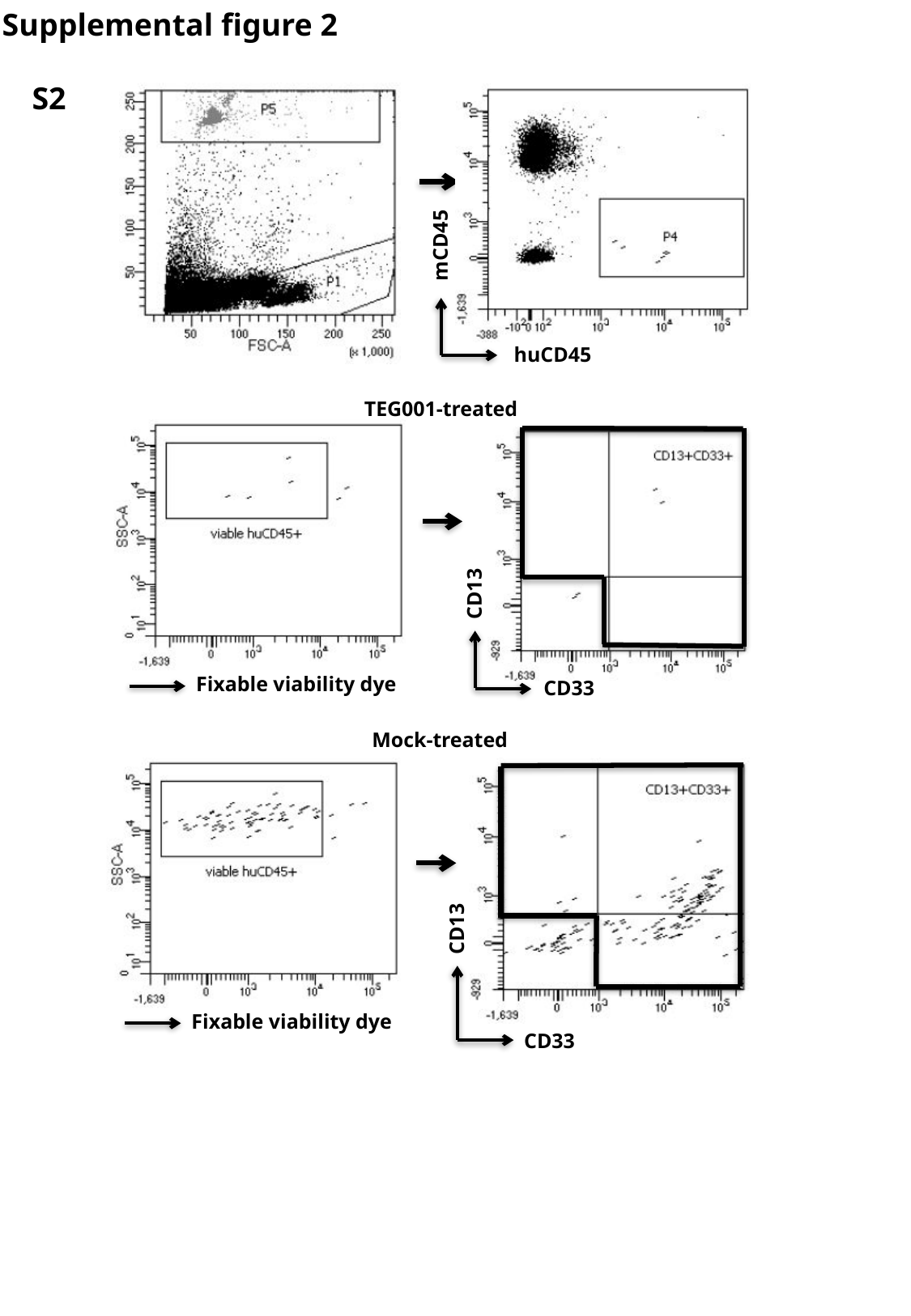

Supplemental figure 2
S2
mCD45
huCD45
TEG001-treated
CD13
Fixable viability dye
CD33
Mock-treated
CD13
Fixable viability dye
CD33
