# Supplementary material for: Evaluating in vivo efficacy – toxicity profile of TEG001 in humanized mice xenografts against primary human AML disease and healthy hematopoietic cells
Source: J Immunother Cancer. 2019 Mar 12;7:69. doi: 10.1186/s40425-019-0558-4 (PMC6419469; doi:10.1186/s40425-019-0558-4)
Supplement: Supplementary file 4 — Figure S1. γδTCR expression of TEG001 and TEG-LM1 mock. A representative flow cytometry plot γδTCR expression of TEG001 and TEG-LM1 mock after transductions, after αβTCR depletion and prior to infusion into mice after 2 weeks expansion. (PPTX 191 kb) [file 40425_2019_558_MOESM4_ESM.pptx]

## Slide 1
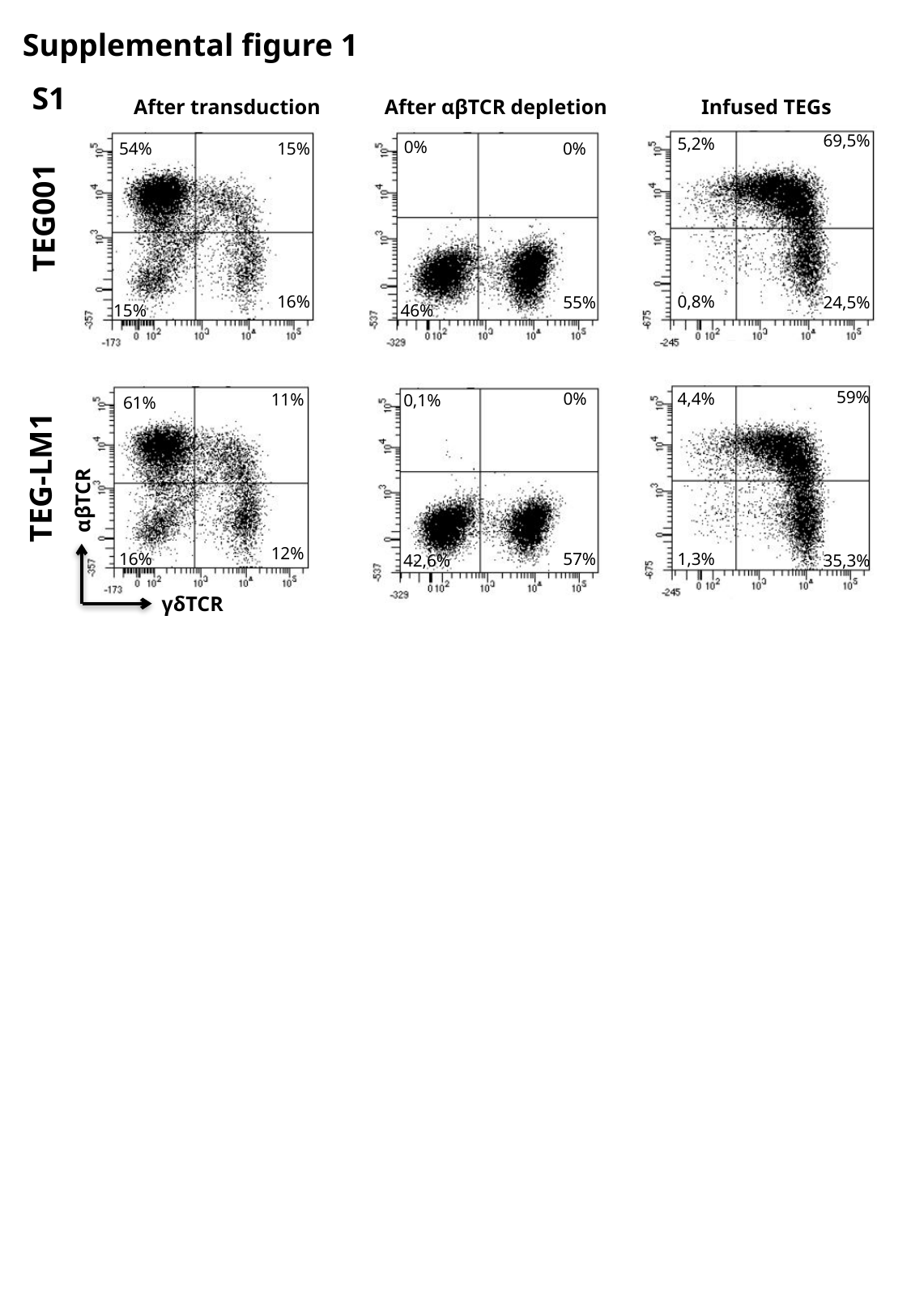

Supplemental figure 1
S1
Infused TEGs
After transduction
After αβTCR depletion
69,5%
5,2%
0%
54%
15%
0%
TEG001
16%
0,8%
55%
24,5%
15%
46%
59%
0%
4,4%
11%
0,1%
61%
TEG-LM1
αβTCR
12%
16%
57%
1,3%
42,6%
35,3%
γδTCR
