# Supplementary figures and images for: Evaluating in vivo efficacy – toxicity profile of TEG001 in humanized mice xenografts against primary human AML disease and healthy hematopoietic cells
Source: J Immunother Cancer. 2019 Mar 12;7:69. doi: 10.1186/s40425-019-0558-4 (PMC6419469; doi:10.1186/s40425-019-0558-4)

## Slide 1
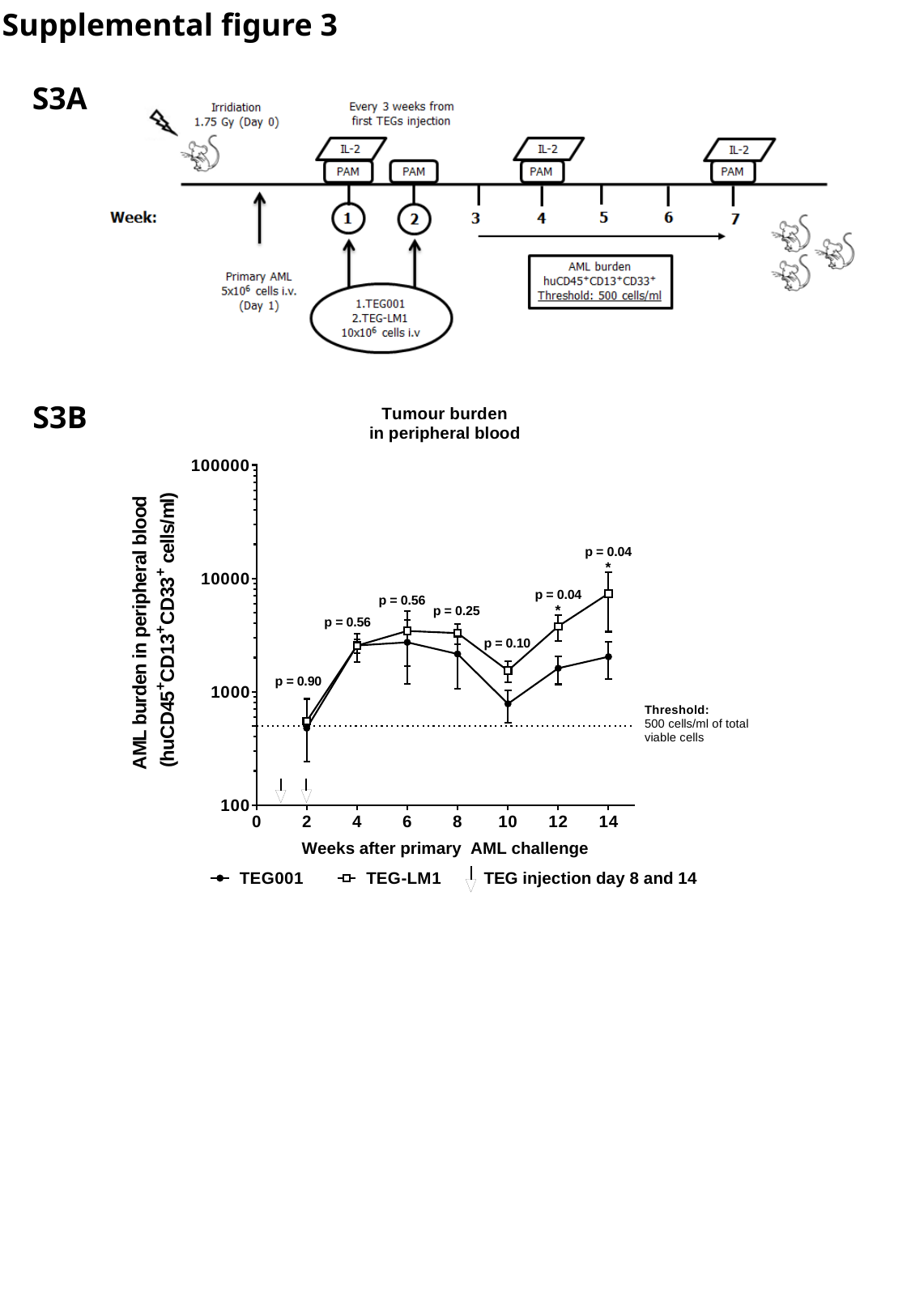

Supplemental figure 3
S3A
S3B

Supplement: Supplementary file 5 — Figure S3. In vivo efficacy profile of TEG001 in PD-X model of primary blast in NSG-SGM3 mice. (A) Schematic overview of in vivo experiment. NSG-SGM3 mice were irradiated at day 0 and engrafted with primary AML cells at day 1. AML cells were followed-up in the peripheral blood by flow cytometry. Mice received 2 injections of therapeutic TEG001 or TEG-LM1 mock in the presence of PAM (at Day 8 and 16) and IL-2 (at Day 8); (B) Tumor burden for primary AML was measured in peripheral blood by quantifying for absolute cell number by flow cytometry. Data represent mean ± SD of all mice per group (n = 5 mice/group). Statistical significances were calculated by non-parametric 2-tailed Mann-Whitney t-test; *, P < 0.05; **, P < 0.01; ***, P < 0.001; ****, P < 0.0001. (PPTX 101 kb) [file 40425_2019_558_MOESM5_ESM.pptx]

## Slide 1
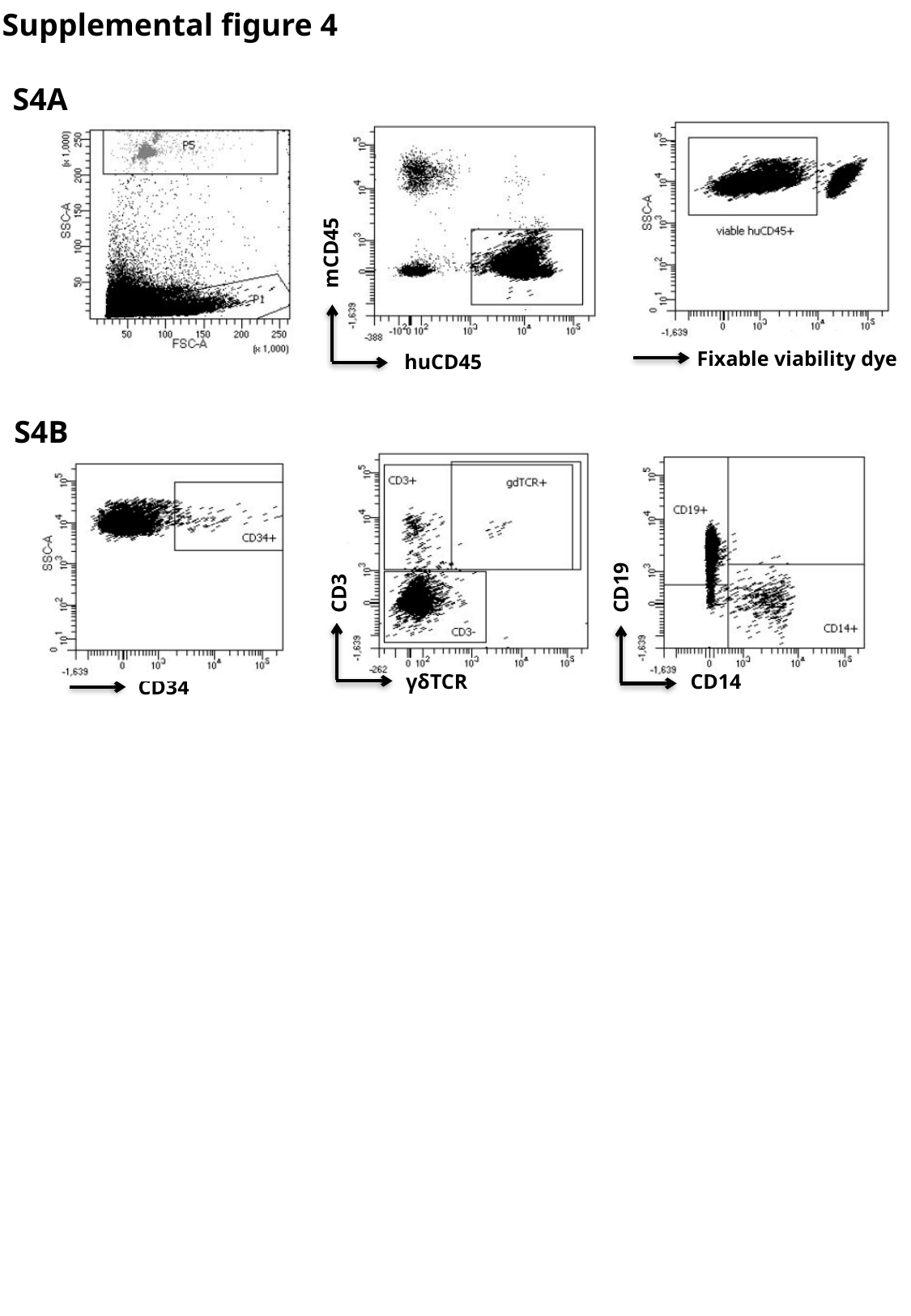

Supplemental figure 4
S4A
mCD45
Fixable viability dye
huCD45
S4B
CD19
CD3
γδTCR
CD14
CD34

Supplement: Supplementary file 6 — Figure S4. Gating strategy for flow cytometry analysis of healthy hematopoietic compartments. A representative flow cytometry plot of murine peripheral blood. (A) Engraftment was determined by quantifying absolute cell number of viable huCD45+ of healthy stem cells; (B) Hematopoietic cellular compartments outgrowth were determined by quantifying absolute cell number for CD19+ B cells, CD3+ T cells, and CD14+ monocytes. Also, persistence of TEGs were determined by quantifying absolute cell number for γδTCR+ cells. (PPTX 225 kb) [file 40425_2019_558_MOESM6_ESM.pptx]
